# Effects of kidney perfusion on renal stiffness and tissue fluidity measured with tomoelastography in an MRI-compatible *ex vivo* model

**DOI:** 10.3389/fbioe.2023.1236949

**Published:** 2023-11-09

**Authors:** Johannes Castelein, Carolina Pamplona, Roberto Armstrong Junior, Marina Vidal dos Santos, Ingolf Sack, Rudi Dierckx, Cyril Moers, Ronald Borra

**Affiliations:** ^1^ Department of Radiology & Nuclear Medicine and Molecular Imaging, University Medical Center Groningen, Groningen, Netherlands; ^2^ Department for Biomedical Sciences, Faculty of Health and Medical Sciences, University of Copenhagen, Copenhagen, Denmark; ^3^ Department of Surgery, University Medical Center Groningen, Groningen, Netherlands; ^4^ Department of Radiology, Charité University Medicine Berlin, Berlin, Germany

**Keywords:** magnetic resonance elastography, tomoelastography, stiffness, fluidity, *ex vivo* kidney, tissue perfusion

## Abstract

Stiffness plays a vital role in diagnosing renal fibrosis. However, perfusion influences renal stiffness in various chronic kidney diseases. Therefore, we aimed to characterize the effect of tissue perfusion on renal stiffness and tissue fluidity measured by tomoelastography based on multifrequency magnetic resonance elastography in an *ex vivo* model. Five porcine kidneys were perfused *ex vivo* in an MRI-compatible normothermic machine perfusion setup with adjusted blood pressure in the 50/10–160/120 mmHg range. Simultaneously, renal cortical and medullary stiffness and fluidity were obtained by tomoelastography. For the cortex, a statistically significant *(p < 0.001)* strong positive correlation was observed between both perfusion parameters (blood pressure and resulting flow) and stiffness (*r = 0.95, 0.91*), as well as fluidity (*r = 0.96, 0.92*). For the medulla, such significant (*p < 0.001)* correlations were solely observed between the perfusion parameters and stiffness (*r = 0.88, 0.71*). Our findings demonstrate a strong perfusion dependency of renal stiffness and fluidity in an *ex vivo* setup. Moreover, changes in perfusion are rapidly followed by changes in renal mechanical properties—highlighting the sensitivity of tomoelastography to fluid pressure and the potential need for correcting mechanics-derived imaging biomarkers when addressing solid structures in renal tissue.

## Introduction

Renal fibrosis, triggered by an initial injury, is the ultimate hallmark of many chronic kidney diseases (CKD) (Liu, 2006; Hewitson, 2012). Regardless of the underlying cause, CKD is characterized by progressive renal scarring that ultimately affects all structures of the kidney as well as renal function. The relentless progression of CKD is postulated to result from a self-perpetuating vicious cycle of fibrosis activated after the initial injury, which can ultimately lead to end-stage renal failure requiring dialysis or kidney transplantation (Fogo, 2007; Anders et al., 2018). This cycle of fibrosis is characterized by the excess accumulation of extracellular matrix causing increased renal stiffness (Wynn and Ramalingam, 2012; Gandhi et al., 2019).

An invasive kidney biopsy is the diagnostic gold standard to measure renal fibrosis (Hysi and Yuen, 2020). Despite the risks of bleeding and sampling bias—at best less than 1% of the kidney is analyzed using this approach (Williams et al., 2012)—the histological evaluation of a kidney biopsy currently depicts the only clinical tool for nephrologists to assess fibrosis (Mansour et al., 2017). Other blood and urine based biomarkers such as serum creatinine (SCr) and albumin excretion rate (AER) are biased by dietary intake and many other factors and are not sufficiently specific to distinguish CKD from other kidney diseases (Lopez-Giacoman, 2015; Greenberg et al., 2018). Hence, there is an urgent need for improved non-invasive diagnostics to quantify renal fibrosis and its consequences in the kidney (Hysi and Yuen, 2020).

Magnetic resonance elastography (MRE) is an advanced quantitative magnetic resonance imaging (MRI) technique that is gaining popularity in diagnosing renal diseases (Venkatesh and Ehman, 2015; Kirpalani et al., 2017). It can detect changes in the viscoelastic properties of soft tissues, which occur during pathological processes (Venkatesh and Ehman, 2015; Kolipaka et al., 2016). Tomoelastography is a recently introduced MRE technique providing shear wave speed and phase angle of the complex shear modulus as surrogate markers of tissue stiffness and fluidity with high spatial fidelity (Tzschätzsch et al., 2016). Tissue stiffness is a measure of the resistance of tissue to deformation when a force is applied. Tissue fluidity reflects the ability of a tissue to deform plastically, to generate friction and absorb mechanical energy during deformation. Tissue fluidity is so named because it ranges from zero (elastic solid) to values found in pure liquids.

In contrast to traditional single-frequency MRE, which is currently used clinically for non-invasive assessment of liver fibrosis, tomoelastography uses wave fields with vibrations of multiple frequencies (Dittmann et al., 2017) being consecutively administered to the tissue. This multifrequency approach improves anatomical resolution, noise robustness, and intra-tissue homogeneity (Tzschätzsch et al., 2016). Previous studies have demonstrated the diagnostic accuracy of tomoelastography to quantify renal stiffness in different cohorts (Marticorena Garcia et al., 2018a; Marticorena Garcia et al., 2019; Marticorena Garcia et al., 2021). Moreover, correlations between renal stiffness in allografts and clinical markers such as glomerular filtration rate (GFR) and resistive index (RI) have been reported (Garcia et al., 2016). Despite these promising results, growing evidence indicates the influence of perfusion on tissue stiffness in addition to fibrosis (Warner et al., 2011; Gennisson et al., 2012; Yin et al., 2017; Gandhi et al., 2019; Brown et al., 2020). Garcia *et al.* (Marticorena Garcia et al., 2018b) showed increasing stiffness in the *ex-vivo* porcine renal cortex resulting from rising vascular pressure. However, since in this study, the vascular pressure was artificially altered through water inflow into the renal vein instead of physiological modulation of blood perfusion, the results may change in a more realistic scenario of kidney perfusion. Specifically, the relationship between MRE parameters and amount of renal perfusion needs to be studied in a controlled scenarion to understand and potentially correct perfusion effects on *in vivo* tomoelastography in the future.

Renal normothermic machine perfusion (NMP) is a revolutionary method for evaluating *ex vivo* kidney graft quality. This technique offers the possibility of creating an isolated near-physiological environment by circulating a perfusion solution at body temperature (35°C–37°C) together with oxygen and nutrients. The potential of NMP-derived markers in assessing the quality of a kidney prior to transplantation has been shown by different research groups (Hosgood et al., 2018; Kaths et al., 2018; Woud et al., 2019; Rigalli et al., 2020). However, due to varying NMP protocols and approaches, it is still unclear which biomarkers during perfusion can reliably indicate the organ’s viability *ex vivo* (Dittmann et al., 2016).

In this study, we investigated whether blood pressure and flow influence the stiffness and fluidity in the cortex and medulla of *ex vivo* perfused porcine kidneys. To answer this question, we assessed the shear wave speed and phase angle of the shear modulus in five porcine kidneys, perfused *ex vivo* in an MRI-compatible NMP circuit with various blood pressures and flows. To the best of our knowledge, this is the first study to evaluate the dependency of perfusion on renal mechanical properties in NMP *ex vivo* kidneys using tomoelastography.

## Materials and methods

### Organ procurement and renal NMP

Five viable porcine kidneys were retrieved *en bloc* 20 min after cardiac arrest from three healthy pigs (Dutch landrace pigs, around 130 kg) in a local slaughterhouse in accordance with all guidelines of the Dutch food safety authority. Subsequently, kidneys were preserved using a pressure-controlled oxygenated hypothermic (1°C–10°C) machine perfusion (O-HMP) (Kidney Assist Transport^®^, XVIVO, Göteborg, Sweden), primed with cold University of Wisconsin Machine Perfusion Solution (Belzer MPS^®^, Bridge to Life, Columbia, United States), at a pressure of 25 mmHg. Due to the time interval between organ procurement and MRI scanner availability, the kidneys were preserved using O-HMP for 10 h with an oxygen flow rate of 0.1 L/min, mimicking clinical cold organ preservation.

**FIGURE 1:**
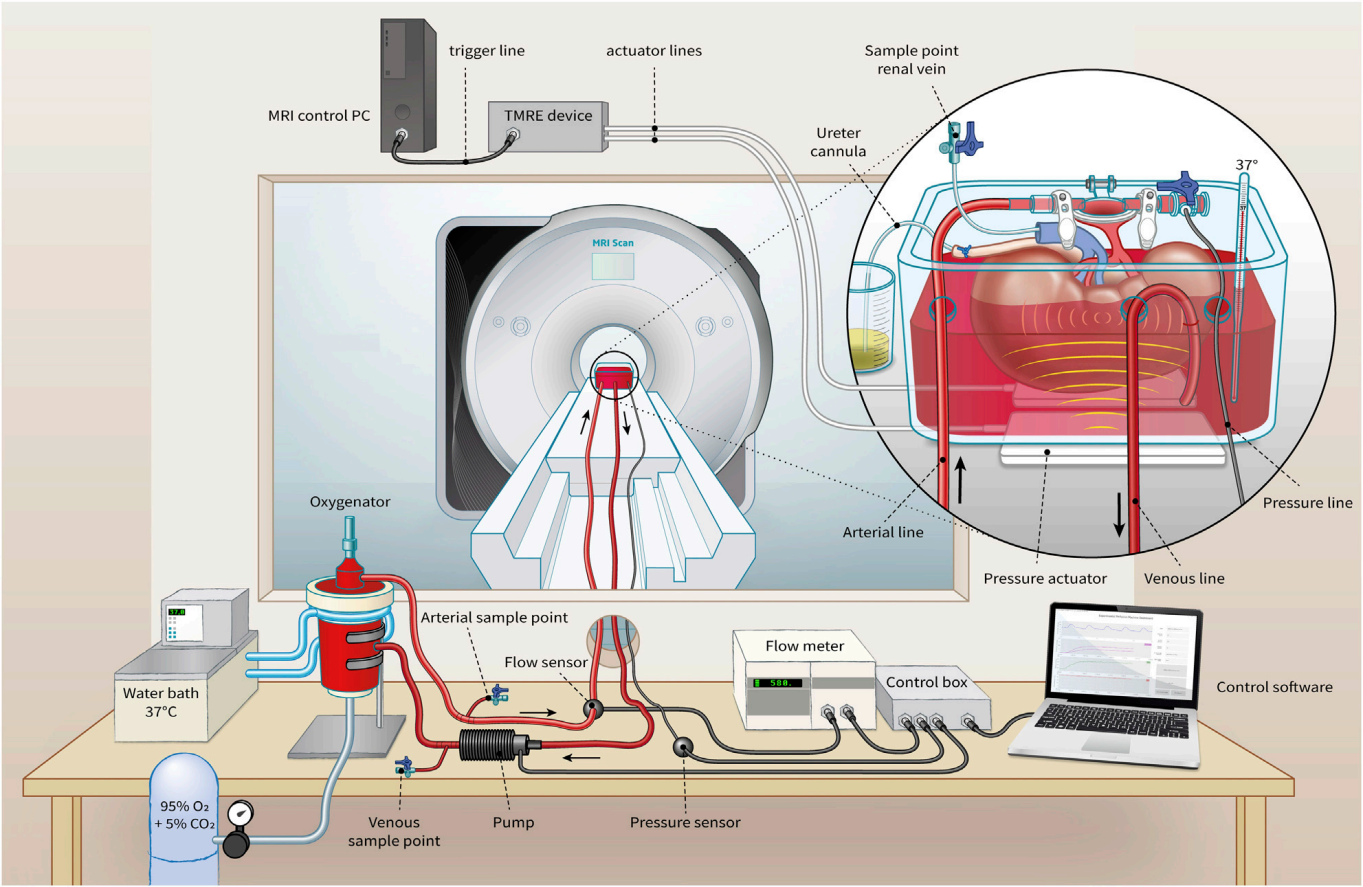
Representation of MRI-compatible NMP setup for *ex vivo* kidney perfusion including Tomoelastography setup. Pressure actuators induce compression wave (yellow) to the organ chamber, evoking shear waves (light red) in the kidney.

At least 1 hour prior to the tomoelastography scan, each kidney was separately perfused with a solution composed of isolated autologous red blood cells (RBCs), crystalloids, albumin, creatinine, antibiotics, and electrolyte supplementation at 35°C–37°C in an MRI compatible NMP circuit (see Figure 1). Oxygenation was administered with carbogen (95% O_2_/5% CO_2_) at a rate of 0.5 L/min using a clinical-grade oxygenator (Hilite 800 LT, Medos Medizintechnik AG, Stolberg, Germany). The kidney was positioned inside a custom-built organ chamber (LifePort^®^, Organ Recovery Systems, Itasca, IL, United States) in the isocenter of the MRI scanner and connected to the other perfusion hardware in the scanner’s control room with 7.5 m long polyvinylchloride tubing.

Blood pressure was controlled through a centrifugal pump (Medos Medizintechnik AG, Stolberg, Germany) using custom-made hard- and software (LabView Software, National Instruments Netherlands B.V., Woerden, the Netherlands). Perfusion pressure was continuously measured in the arterial cannula through four connected pressure lines (Lectro-Cath V-Green PE, Vygon, Ecouen, France) attached to a clinical-grade pressure sensor (TruWave disposable pressure transducer, Edwards Lifesciences, Irvine, CA, United States) positioned in the scanner’s control room at the same height as the kidney. The following systolic/diastolic pressure scenarios were set: 160/120, 110/70, 80/40, and 50/10 mmHg with a pause of 1 minute in between to allow the kidney to adapt to the change in tissue perfusion. Resultant renal flow rates were externally measured on the arterial line with an ultrasonic flow sensor (Transonic^®^ HQXL Flowsensors, Ithaca, IL, United States).

### *Ex vivo* tomoelastography

Tomoelastography was performed utilizing two pneumatic actuators which were placed under the organ chamber. The compressed air-driven actuators induced four vibration frequencies in the range of 40–70 Hz with an increment of 10 Hz and air pressure of 650–800 mbar. All examinations were performed on a 3 T MRI system (Magnetom Prisma; Siemens Healthineers, Erlangen, Germany) using a 20-channel phased-array head coil. The acquisition of the 3-D wave fields was based on the one proposed by Dittmann *et al.* (Dittmann et al., 2016) using a single-shot, spin-echo planar imaging sequence with flow-compensated motion-encoding gradients (MEG). Eight wave-phase offsets were recorded for each of the three motion directions. In total, 19 paracoronal slices with 2 mm isotropic resolution covering the entire *ex vivo* kidney were acquired in 4 minutes. Other tomoelasography parameters were: TR = 2,260 ms; TE = 69 ms; FOV of 256 × 256 mm^2^ (matrix size: 128 × 128) parallel imaging with a GRAPPA factor of 2; MEG frequency = 37.20 Hz, 37.48 Hz, 45.13 Hz, and 74.40 Hz for vibration frequencies of 40 Hz, 50 Hz 60 Hz, and 70 Hz; MEG amplitude = 40 mT/m.

### Data processing

**FIGURE 2:**
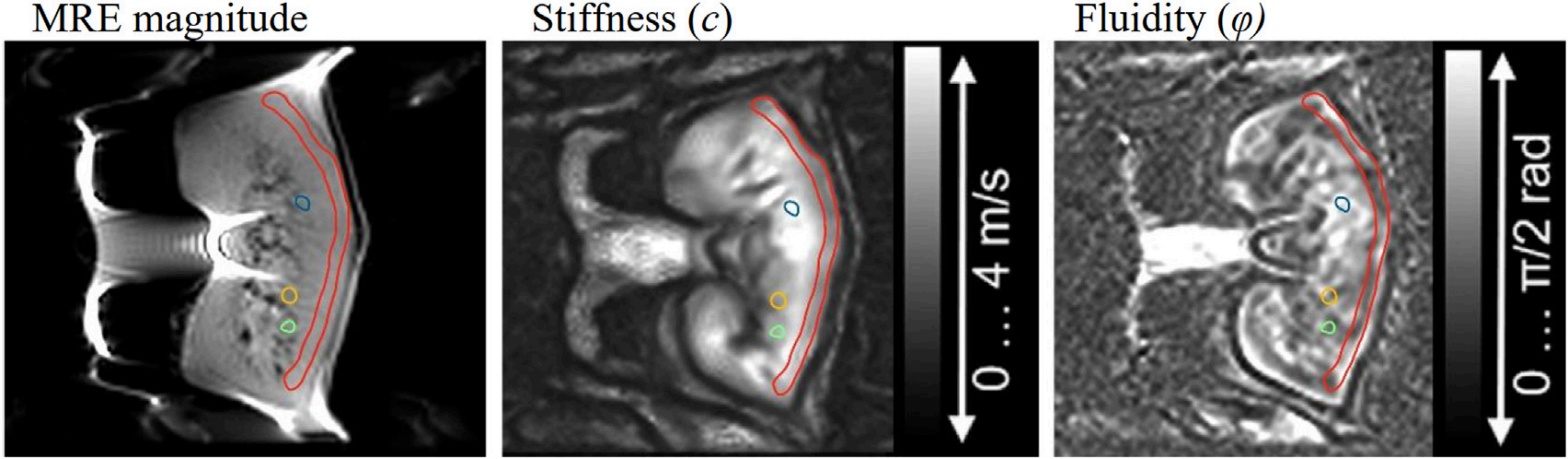
Example of cortical (red) and medullary (blue, yellow, green) segmentation for the analysis of stiffness and fluidity.

Tomoelastography data were post-processed using multifrequency dual elasto-visco inversion programs publicly available at https://bioqic-apps.charite.de(Meyer et al., 2022). Shear wave speed (*c* in m/s) was retrieved from multifrequency wave-number recovery (k-MDEV) inversion algorithm and the phase angle of the complex shear modulus, *φ* (range: *0–π/2*) from the multifrequency direct inversion (MDEV). Shear wave speed reflects tissue stiffness as it is derived from the real part of wave numbers. Furthermore, *φ* is an indicator for the solid–fluid behavior of the tissue and is referred to as fluidity for fluid dominated tissue properties (*φ* > *π/4*). Pre-smoothing was performed using a Gaussian kernel for both inversion algorithms. Additionally, to suppress low-frequency compression waves, a linear highpass in k-space was employed for k-MDEV and a bandpass Butterworth filter of third order for MDEV. More details concerning the applied inversion methods are given in Streitberger *et al.* (Streitberger et al., 2014a) for MDEV and Tzschätzsch *et al.* (Tzschätzsch et al., 2016) for k-MDEV. The resulting elastograms reflecting tissue stiffness (*c*) as well as tissue fluidity (*φ*) were visualized with open‐source DICOM viewer Horos^®^ (version 3.3.6, https://horosproject.org/). Based on paracoronal anatomical T2-weighted images of the MRE scan, regions of interest (ROIs) were manually drawn in the renal cortex and three different medullary pyramids. Example ROIs are shown in Figure 2.

### Statistical analysis

All values are expressed as means ± standard deviations. The Shapiro–Wilk test was used to check the normality assumption for quantitative variables. Pearson’s correlation coefficient was calculated for bivariate correlation to investigate the relationship between mechanical properties (stiffness and fluidity) and perfusion (blood pressure and flow). All statistical analyses and visualizations were performed using OriginPro, (Version 2022b; OriginLab Corporation, Northampton, MA, United States). Statistical significance was assumed when the *p*-value was <0.01.

## Results

Obtained stiffness and fluidity values, as well as tracked blood flow of all *ex vivo* kidneys for the four adjusted blood pressure scenarios, are summarized in Table 1. Overall, stiffness and fluidity increased with blood pressure in the renal cortex and the medulla. In cortical tissue, wave speed increased by approximately 124% and fluidity by 132%, ranging from 50/10 mmHg to 160/120 mmHg. In contrast, enhancement of the medullary mechanical properties was less pronounced, with an approximate increase of 60% in stiffness and 21% in fluidity.

**TABLE 1:** Stiffness, fluidity, and blood flow values for the four adjusted blood pressure scenarios.

Systole/Diastole [mmHg]	Blood flow [mL/min]	Stiffness *c* [m/s]		Fluidity *φ* [rad]	
		Cortex	Medulla	Cortex	Medulla
50/10	214.61 ± 24.0	1.47 ± 0.16	2.02 ± 0.23	0.55 ± 0.06	0.89 ± 0.27
80/40	333.46 ± 84.80	1.88 ± 0.25	2.30 ± 0.17	0.68 ± 0.04	0.91 ± 0.19
110/70	466.93 ± 99.44	2.43 ± 0.29	2.85 ± 0.40	0.99 ± 0.09	1.09 ± 0.11
160/120	637.36 ± 109.91	3.30 ± 0.30	3.25 ± 0.23	1.28 ± 0.12	1.08 ± 0.14

**FIGURE 3:**
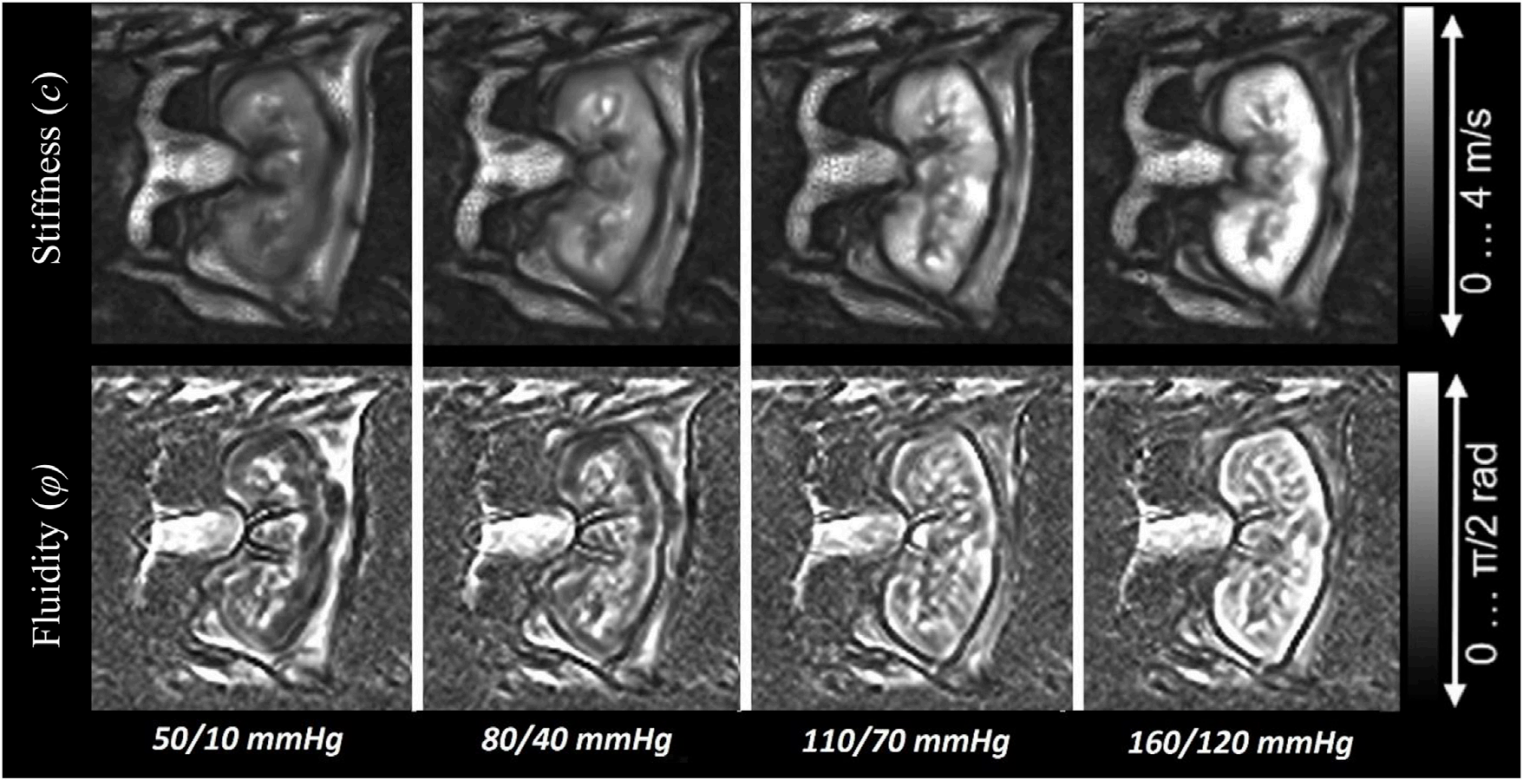
Images illustrating the increase of stiffness and fluidity in the *ex vivo* porcine kidney with rising blood pressure.

Figure 3 shows the visually evident effect of increasing blood pressure on the stiffness and fluidity elastograms.

**FIGURE 4:**
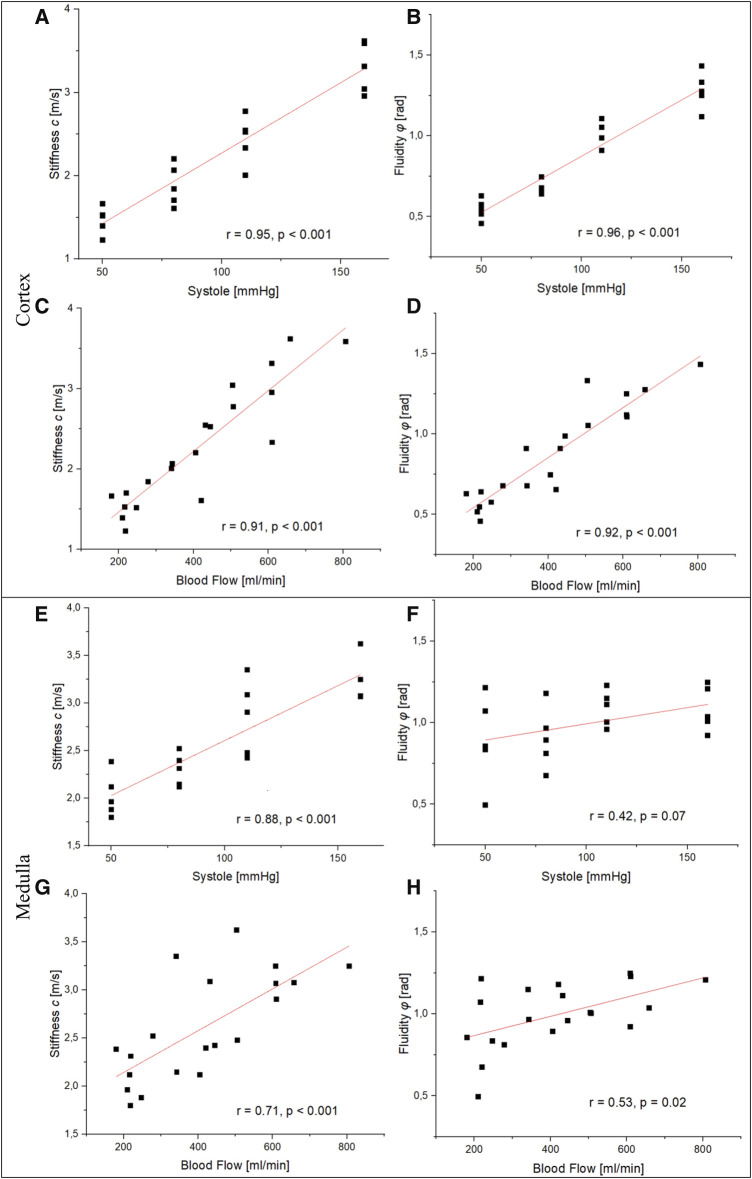
Relationship between perfusion markers and the mechanical properties of the renal cortex and medulla. **(A, B)**, **(C)**, and **(D)** show a statistically significant high positive correlation between blood pressure/flow and stiffness as well as fluidity in the renal cortex. Resemblant significant correlations in the renal medulla were observed between blood pressure/flow and stiffness **(E, G)**. No statistically significant correlation was observed between systolic blood pressure/flow and fluidity **(F)**, **(H)**.

Pearson’s correlation was used to compare perfusion markers with observed tomoelastography findings. Figure 4 shows scatterplots and resulting Pearson correlation coefficients between systolic blood pressure/flow and mechanical parameters in the renal cortex and medulla. We found statistically significant high positive correlations between systolic blood pressure and blood flow in cortical stiffness (blood pressure: *r = 0.95*, *p < 0.001*; blood flow: *r = 0.91*, *p < 0.001*) and fluidity (blood pressure: *r = 0.96*, *p < 0.001*; blood flow: *r = 0.92*, *p < 0.001*). Likewise, we observed a statistically significant association between medullary stiffness and systolic blood pressure (*r = 0.88*, *p < 0.001*) and blood flow (*r = 0.71*, *p < 0.001*). On the other hand, there was no statistically significant correlation between medullary fluidity and systolic blood pressure (*r = 0.42*, *p < 0.07*) nor blood flow (*r = 0.53*, *p < 0.02*). Hence, our results suggest a strong linear relationship between tissue perfusion and renal stiffness as well as cortical fluidity.

## Discussion

Tomoelastography allows early detection of changes in renal stiffness as a result of various kidney pathologies. However, variations in tissue perfusion strongly impact the assessment of renal stiffness (Warner et al., 2011). In the present study, we have characterized the influence of tissue perfusion on stiffness and fluidity in an *ex vivo* perfused porcine kidney model. Our most remarkable finding is the strong positive correlation between increasing vascular pressure (and consequently blood flow) and stiffness in the cortex and medulla of *ex vivo* perfused kidneys. Another promising finding was the rapid change in renal tissue stiffness and fluidity after only 1 minute of altered tissue perfusion.

The entire kidney is surrounded by a fibrous capsule composed of tough fibers with minor flexibility. Increasing vascular pressure leads to an increase in renal cortical and medullary stiffness due to the minimal elasticity of the renal capsule. As the kidney is one of the most highly vascularized organs in the body, variations in perfusion will have a greater impact on the mechanical properties than in other organs. This effect may play a major role in MRE of *in vivo* kidneys to detect early structural changes, as well as to provide valid cut-off values for specific pathologies. It has been discussed that perfusion changes provide the sensitivity to renal MRE to detect glomerulonephritis in patients with preserved renal function (Grossmann et al., 2019). As such, MRE can detect subtle changes in renal tissue softening that may indicate impaired tissue perfusion and reduced fluid pressure at very early stages, making it a sensitive marker for evaluating glomerulonephritis (Marticorena Garcia et al., 2018c; Lang et al., 2019). Otherwise, a correction factor based on blood pressure or blood flow may increase the sensitivity of tomoelastography to solid matrix structures and lead to higher accuracy in inter-study comparisons. Moreover, our findings appear to be similar to the viscoelastic response of the human brain due to altered cerebral perfusion induced by the Valsalva maneuver (Herthum et al., 2021).

During acute kidney injury (AKI), the kidneys undergo functional and structural changes, including inflammation, ischemia, necrosis, and a reduction in regional renal oxygen delivery (Ferenbach and Bonventre, 2016; Hsu and Hsuyuan, 2016; Hatakeyama et al., 2018). AKI and CKD are closely linked and can promote each other (Zhu et al., 2022). In the transition from AKI to CKD, the repair mechanisms in the kidney become maladaptive (Ferenbach and Bonventre, 2016). Instead of fully repairing the damage, the kidneys undergo accelerated fibrosis, which involves excessive collagen deposition and other extracellular matrix components. This fibrotic process contributes to the stiffening of renal tissue and impairs kidney function. However, due to the significant impact of perfusion on renal stiffness, we see an overall renal softening caused by reduced perfusion as a consequence of vessel rarefication. Our results support the theoretical model of renal softening in dysfunctional kidneys due to reduced perfusion, as proposed by Lang *et al.* (Lang et al., 2019). Additionally, our outcomes underline the potential of flow-based correction factors to unmask renal stiffening attributable to fibrosis.

Renal blood flow is well autoregulated. The kidney distributes about 80% of its blood flow to the cortex and 15% to the outer medulla (Duke, 2011). This difference in perfusion between the cortex and medulla is confirmed by the higher cortical stiffness (124% vs. 60% in the medulla) and fluidity (132% vs. 21%) observed in this study. In addition, our results underline the high elastic modulus—indicating a high resistance to deformation—of the fibrous renal capsule. Although Garcia *et al.* (Marticorena Garcia et al., 2018b) have also reported increased cortical stiffness with increased perfusion, their results showed a reduction in medullary stiffness with rising venous inflow pressure. This decrease opposes the generally positive correlation between perfusion and stiffness. Nevertheless, it should be noted that their study was conducted on a single non-perfused porcine kidney, hence not under near-physiological conditions. An *in vivo* study by Gandhi *et al.* (Gandhi et al., 2020) using single-frequency MRE on healthy subjects found a significant increase in renal stiffness after water intake. These results are consistent with our findings. In addition, the fact that we could directly and reliably measure renal blood pressure corroborates the theory that a higher perfusion pressure in the kidney causes an increase in renal stiffness.

Acquired cortical stiffness (*c* = 2.43 ± 0.29 m/s) and medullary stiffness (*c* = 2.85 ± 0.40 m/s) at a blood pressure of 110/70 mmHg—which can be assumed as resting blood pressure—are in line with reported values for *in vivo* porcine kidneys (Warner et al., 2011) of 2.72 m/s at 120 Hz (shear modulus of 7.4 kPa) for the cortex and 2.79 m/s at 120 Hz (shear modulus of 7.8 kPa) for the medulla. Likewise, cortical stiffness is consistent with previous tomoelastography findings in native human kidneys (*c* = 2.52 ± 0.11 m/s Marticorena Garcia et al., 2018a, 2.46 ± 0.25 m/s Shahryari et al., 2021). However, we measured higher medullary stiffness compared to the previous study (*c* = 2.15 m/s ± 0.08 m/s) Marticorena Garcia et al., 2018a. In addition, tissue fluidity, expressed by the phase angle in radians, in the cortex (*φ* = 0.99 ± 0.09) and medulla (*φ* = 1.09 ± 0.11) was slightly higher than reported by Streitberger *et al.* (Streitberger et al., 2014b) (*φ*_*Cortex*_ = 0.83 ± 0.09, *φ*_*Medulla*_ = 0.89 ± 0.12). We attribute this discrepancy to the different experimental setup, as (Streitberger et al., 2014b) performed an *in vivo* assessment compared to our *ex vivo* approach. The difference here could be attributed to the lacking innervation of *ex vivo* kidney, thus lacking autoregulation of renal blood flow. Especially fluidity could be more directly influenced by vascular interaction when the kidney is not autoregulated (Gandhi et al., 2020; Shahryari et al., 2021). By all means, our study contributes to unraveling factors that aid in detecting renal structural changes.

Our study has several limitations. First, the slight observer-dependency of the manual ROI segmentation that we performed may have affected the quantification of mechanical parameters. This especially holds true for the porcine medulla, which is smaller than a human kidney, making proper delineation challenging. Second, we quantified the total renal blood flow and correlated it to regional stiffness measures of the cortex and medulla. The additional assessment of regional tissue perfusion using MRI perfusion imaging techniques such as dynamic susceptibility contrast (DSC) and arterial spin labeling (ASL) would have enabled a more sophisticated approach to explore the relationship between renal perfusion and stiffness. Thirdly, *ex vivo* perfused kidneys are abnormally innervated, leading to lacking autoregulation of renal blood flow (Shannon et al., 1998). Hence, high arterial blood pressures are directly transferred to the graft endothelium, making the stiffness of *ex vivo* kidneys much more closely related to perfusion pressure than native kidneys. As demonstrated by Garcia *et al.* (Garcia et al., 2016), dysfunctional allografts are softer than their functional counterparts, further highlighting the sensitivity of MRE in detecting changes in renal perfusion. Finally, there were variations in the duration of O-HMP and NMP prior to the tomoelastography scan (1–5.5 h), which might have influenced intrarenal flow distribution.

In conclusion, our study emphasizes the impact of perfusion on the mechanical properties of renal tissue and highlighted the sensitivity of tomoelastography to fluid pressure. Moreover, we observed that renal stiffness and fluidity rapidly change when renal hemodynamics are altered. Therefore, future studies should focus on validating our results in an *in vivo* setting and determining correction factors for regional tissue perfusion in the renal cortex and medulla.

## Data Availability

The raw data supporting the conclusion of this article will be made available by the authors, without undue reservation.
